# Synchrotron X-Ray Radiation-Induced Bystander Effect: An Impact of the Scattered Radiation, Distance From the Irradiated Site and p53 Cell Status

**DOI:** 10.3389/fonc.2021.685598

**Published:** 2021-05-21

**Authors:** Pavel Lobachevsky, Helen B. Forrester, Alesia Ivashkevich, Joel Mason, Andrew W. Stevenson, Chris J. Hall, Carl N. Sprung, Valentin G. Djonov, Olga A. Martin

**Affiliations:** ^1^ Research Division, Peter MacCallum Cancer Centre, Melbourne, VIC, Australia; ^2^ Advanced Analytical Technologies, Melbourne, VIC, Australia; ^3^ Centre for Innate Immunity and Infectious Diseases, Hudson Institute of Medical Research, Clayton, VIC, Australia; ^4^ Department of Molecular and Translational Science, Monash University, Clayton, VIC, Australia; ^5^ School of Science, Royal Melbourne Institute of Technology (RMIT) University, Melbourne, VIC, Australia; ^6^ Therapeutic Goods Administration, Canberra, ACT, Australia; ^7^ Florey Institute of Neuroscience and Mental Health, Melbourne, VIC, Australia; ^8^ Commonwealth Scientific and Industrial Organisation (CSIRO) Future Industries, Clayton, VIC, Australia; ^9^ Australian Nuclear Science and Technology Organisation (ANSTO)/Australian Synchrotron, Clayton, VIC, Australia; ^10^ Institute of Anatomy, University of Bern, Bern, Switzerland; ^11^ Department of Radiation Oncology, Peter MacCallum Cancer Centre, Melbourne, VIC, Australia; ^12^ University of Melbourne, Melbourne, VIC, Australia

**Keywords:** synchrotron radiation, microbeam radiation therapy (MRT), scattered radiation, radiation induced bystander effect (RIBE), DNA damage, gamma-H2AX (γH2AX), p53

## Abstract

Synchrotron radiation, especially microbeam radiotherapy (MRT), has a great potential to improve cancer radiotherapy, but non-targeted effects of synchrotron radiation have not yet been sufficiently explored. We have previously demonstrated that scattered synchrotron radiation induces measurable *γ*-H2AX foci, a biomarker of DNA double-strand breaks, at biologically relevant distances from the irradiated field that could contribute to the apparent accumulation of bystander DNA damage detected in cells and tissues outside of the irradiated area. Here, we quantified an impact of scattered radiation to DNA damage response in “naïve” cells sharing the medium with the cells that were exposed to synchrotron radiation. To understand the effect of genetic alterations in naïve cells, we utilised p53-null and p53-wild-type human colon cancer cells HCT116. The cells were grown in two-well chamber slides, with only one of nine zones (of equal area) of one well irradiated with broad beam or MRT. *γ*-H2AX foci per cell values induced by scattered radiation in selected zones of the unirradiated well were compared to the commensurate values from selected zones in the irradiated well, with matching distances from the irradiated zone. Scattered radiation highly impacted the DNA damage response in both wells and a pronounced distance-independent bystander DNA damage was generated by broad-beam irradiations, while MRT-generated bystander response was negligible. For p53-null cells, a trend for a reduced response to scattered irradiation was observed, but not to bystander signalling. These results will be taken into account for the assessment of genotoxic effects in surrounding non-targeted tissues in preclinical experiments designed to optimise conditions for clinical MRT and for cancer treatment in patients.

## Introduction

Abscopal, or distant, effects of ionising radiation (IR) were first described by Mole in 1953 (1), and have been regularly documented subsequently (2). This definition refers to non-targeted radiation responses in parts of the body distant from the irradiated volume. The radiation-induced bystander effect (RIBE) is the counterpart *in vitro* phenomenon that describes the effect of IR in non-irradiated (naïve) cells sharing the milieu with targeted cells (3, 4). The implicated mechanisms underlying RIBE involve cell-signalling cascades, release of reactive oxygen/nitrogen species, growth factors, cytokines, and, very recently, exosomes (3, 5, 6). RIBE is thought to be transmitted *via* gap-junction intercellular communication (7), or extracellular soluble factors (8). X-ray beams produced by the third-generation synchrotron source such as Australian Synchrotron (AS) in Melbourne, Australia and the first fourth-generation European Synchrotron Radiation Facility (ESRF) in Grenoble, France, have the advantage of delivering high radiation doses to a very small volume with low beam divergence (9, 10). These features facilitate the study of *in vitro* and *in vivo* non-targeted effects.

Our group has reported non-targeted biological effects in partially irradiated cell populations *in vitro*, and in mouse models at the imaging medical beamline (IMBL) at the AS (11–14). Various biological endpoints were employed in these studies, namely DNA damage response (DDR), apoptotic cell death, oxidative stress, senescence and the immune response. Mothersill’s group has studied non-targeted radiation effects in non-tumour and tumour bearing animals irradiated at the ESRF. Fernandez-Palomo et al. (15, 16) reported non-targeted effects following synchrotron irradiation of tumour-free and tumour-bearing rat brains, occurring within partially irradiated rats and between irradiated and non-irradiated rats caged together. Synchrotron experiments of this group involved clonogenic cell survival, calcium flux, role of 5-hydroxytryptamine (5HT), reporter assay cell death and proteomic profile of non-irradiated organs as end-points (17). Still, there is a lot of research to be done to understand the physical extent of these non-targeted effects, their mechanisms, to minimise the risks to non-irradiated normal tissues, and to simultaneously optimise abscopal anti-tumour effects.

In our previous studies we have employed the *γ*-H2AX assay (18, 19) as a sensitive quantitative tool to detect both substantial and marginal differences in cellular DDR, in particularly, in occurrence and resolution of DNA double-strand breaks (DSBs) in non-targeted cultured cells (20–23), 3-D tissue models (24) and animal organs (12, 25, 26). We have found that, in contrast to the *γ*-H2AX response in directly irradiated cells and tissues, RIBE and abscopal effects are characterised by a delayed peak of *γ*-H2AX foci formation and maintaining unrepaired DSBs for a longer time period. The extent of this delay varies between experimental models, being hours in cultured cells and days in tissue and animal models.

The possible contribution of scattered radiation to observed bystander and abscopal effects has been acknowledged (11), but not thoroughly addressed experimentally. Our radiochromic film dosimetry and *γ*-H2AX-based biodosimetry studies in transformed human keratinocytes FEP1811 have revealed that scattered radiation from both broad beam (BB) and a spatially fractionated beam, or microbeam radiotherapy (MRT) synchrotron radiation, induced *γ*-H2AX foci in a dose-dependent manner, and that the exposure from scattered radiation contributed to the observed RIBE (11). This study also provided a guidance to estimate scatter doses following exposure of biological targets to high dose-rate synchrotron radiation. It is acknowledged that biological effects of scattered radiation, such as accumulation of DNA damage, genomic instability, mutagenesis and ultimately secondary cancers, can follow synchrotron radiotherapy, as it has been reported for conventional radiotherapy and particle irradiation (4, 27).

Here, we investigated a spatio-temporal generation of RIBE, by scoring *γ*-H2AX foci induced by synchrotron BB and MRT radiation in non-irradiated cell cultures. We utilised an experimental system that allowed direct comparison of the level of bystander DNA damage with the level of DNA damage generated by scattered radiation. Finally, we compare DDR in human colon cancer cells bearing p53 wild-type (WT) or p53-null, to further understand a role of this “guardian of the genome” in response to low-dose IR and in propagation of RIBE. This study provides the basis for consequent *in vivo* studies of non-targeted effects of synchrotron RT which could have a profound effect on the planning of cancer MRT regimens.

## Materials and Methods

### Cell Cultures

Human colon cancer cells HCT-116, p53-wild type (WT) or p53-null, generated by targeted disruption of the p53 alleles in parental HCT116 cells, were originally obtained by B. Vogelstein, Johns Hopkins University School of Medicine (28). The cells were a gift to Dr Carleen Cullinane at Peter MacCallum Cancer Centre in Melbourne and then passed to the OAM group. The cells were cultured in Dulbecco’s Modified Eagle Medium (DMEM) complemented with 10% Foetal Bovine Serum (FBS), 2.0 ml-glutamine, 100 U/ml Penicillin and 100 µg/ml Streptomycin (all reagents from Life Technologies, Australia). Cell cultures were maintained at 37°C in a humidified environment of 5% CO_2_. The cells were plated in two-well chamber slides (BD, Franklin Lakes, NJ, USA) and incubated overnight prior to irradiation and irradiated at 75-80% confluency.

### Experimental Set-Up and Irradiations

 Irradiations were conducted at the IMBL, AS, Melbourne. The chamber slides with sealed covers containing adherent cell cultures in growth medium in each well, were oriented with the glass base perpendicular to the beam. The flasks were filled with medium before having a plastic film (Parafilm, Sigma-Aldrich, Australia) placed over the chamber wells. After irradiation the film was removed and the medium volume was reduced to the original volume (1 ml) for cell growth. The original lid for the chamber wells was used for post-irradiation incubation, which allows gas exchange with the surroundings.

The X-ray beam was used for irradiations, with a constant electron current of 200 mA, dose-rate of 49.3 Gy/s and weighted mean photon energy of 94.4 keV (9). The beam dimension was set to 8 mm width and 1 mm height, for both BB and MRT modes. For MRT irradiation, an array of microbeams was produced by placing a collimator in the beam that generated 5 planar 25 µm-wide beams with a 175 µm vertical inter-beam separation. With the aid of a motorized stage, eight consecutive 811 ms beam pulses were applied with a vertical increment of the stage position by 1 mm after each pulse, resulting in 8x8-mm irradiated area and 40 Gy peak-dose in BB and MRT. Given the geometry of the microbeam collimator, it is expected that for an “ideal” microbeam the peak and inter-beam (valley) doses would be 40 and 0 Gy respectively, and the average dose integrated over the whole irradiated area (8x8 mm) would be equal to 1/8 of the peak dose, i.e. 5 Gy. For the real microbeam, however, the valley doses are greater than 0 Gy and peak doses are less than expected 40 Gy, thus the real average integrated dose was not known at the time of the experiment. Therefore, we decided to compare the effect of BB and MRT for equal duration of irradiation (811 ms). This implied, in the context of the study objectives, that the results would allow to establish which factor, peak dose or average dose, determines the extent of RIBE. As it was subsequently calculated and reported in (11), the average integrated dose for MRT irradiations was 4.64 Gy.

Note that the dose rate exceeds the defined for FLASH-RT, initially characterized as using dose-rate >40 Gy/s for conventional radiation (29). Recently however, the biological FLASH-RT effect was found to be reproducible when the whole dose of radiation (the peak-dose in the case of MRT) is delivered in less than 200 milliseconds (30). Therefore, not all MRT sources will be able to have a FLASH effect since the biological FLASH-RT effect, as, for example, the delivery of a peak-dose of 400 Gy requires a dose rate of 2000 Gy/s as a minimum to be delivered in 200 milliseconds. It was not the case in our study, where the dose-rate of 49.3 Gy/s and peak-dose of 40 Gy have been used.

**Figure 1:**
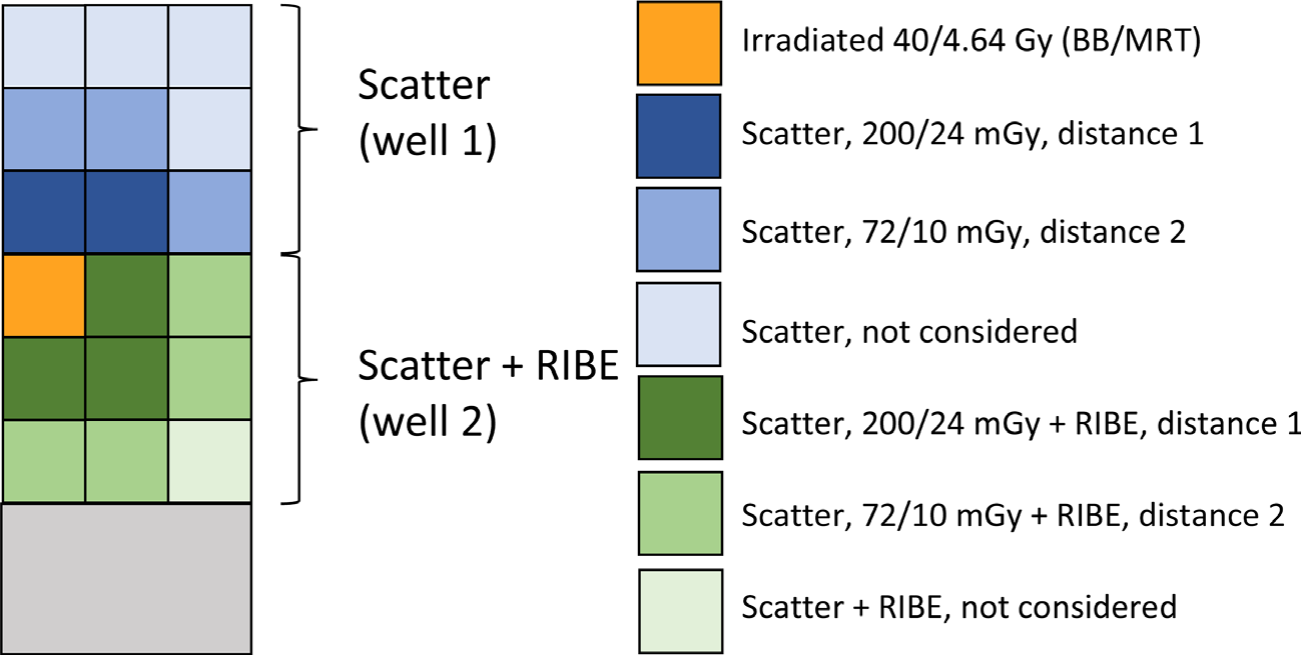
Experimental set-up for BB and MRT irradiations. The image is a schematic presentation of a two-well chamber slide; the chambers are divided by a plastic separator. Each well is considered as nine 8x8mm zones, with only cells in the upper top left zone of well 2 (orange) being irradiated. Well 1 was not irradiated. Because well 1 was physically separated from the irradiated well 2, it was only used for the assessment of DDR induced by scattered radiation. In well 2, irradiated and non-irradiated cells shared the medium, therefore the non-irradiated cells were exposed to both scattered radiation and bystander signalling from the irradiated cells. *γ*-H2AX foci per cell values in cells from the non-irradiated well 1 at different distances from the irradiated site were compared to the commensurate distance values in the irradiated well 2 (distance 1: next to the irradiated area, dark blue and dark green; distance 2: far from the irradiated area, blue and green). The distances that did not have a sufficient match (light blue and light green), were not considered. Average doses delivered by the beam or scattered radiation are shown in right part of the figure for BB and MRT irradiations.

Only one 8x8 mm area was irradiated in well 2 of each slide. Well 1 was not irradiated. Experimental set-up is shown in **Figure 1**. Mock-irradiated reference cells were processed in a similar way, but without irradiation. For each cell line, several variables were used; two irradiation modalities - BB and MRT, three post-irradiation time-points for BB (0.5, 4 and 24 hours) and two time-points for MRT (0.5 and 24 hours).

For a study of a low-dose radiation response, p53 WT and p53-null HCT-116 cells grown in two-well chamber slides were exposed to conventional X-ray irradiations. Irradiations were conducted on an X-RAD iR-160 X-ray source (Precision X-ray Inc., North Branford, CT) operating at 160 kVp, 19 mA with built-in 0.8 mm Be and 2 mm Al filters at a dose rate of 1.87 Gy/min. The cells were irradiated with doses in the range 10 - 1000 mGy) and fixed at 0.5, 4 and 24 hours post-irradiation.

### Immunocytochemistry, Microscopy and Image Analysis

After irradiations, the cells were returned to the cell culture incubator and incubated at 37°C, 5% CO_2_ for indicated times. The cells were fixed for 20 min in 2% paraformaldehyde in phosphate buffered saline (PBS) and processed for immunostaining as described elsewhere (31–33). Briefly, the samples were washed in PBS, blocked for 30 min in 1% bovine serum albumin in PBS-TT (0.5% Tween 20 (Merck KGaA, Darmstadt, Germany), 0.1% Triton X-100 (Sigma-Aldrich, St. Louis, MO, USA), and incubated with primary mouse monoclonal anti-*γ*-H2AX antibody (Abcam, Cambridge, UK), and then with secondary Alexa Fluor 488 goat anti-mouse IgG (Invitrogen, Australia). The slides were mounted with Vectashield mounting medium containing propidium iodide (PI, Vector Laboratories, Burlingame, CA, USA). Laser confocal scanning microscopy was performed using an Olympus FV1000 laser scanning microscope (Olympus, Tokyo, Japan), and images collected for each of the unirradiated zones of interest indicated in **Figure 1**; 5 zones in well 1, and 7 zones in well 2, each corresponding to distances D1 or D2 from the irradiated zone in well 2.

### Data Analysis

Automatic foci counting was performed using the in-house developed JCountPro software, which is an improved version of the TGI software that has been reported previously (34–37).

All images were classified into groups according to the various combinations of the tested experimental variables. These categorical variables included: 1) ‘*Beam*’ (two levels, BB and MRT); 2) ‘*Well*’ (two levels, Well 1 – scatter and Well 2 – scatter and RIBE); 3) ‘*Distance*’ (two levels, distance 1 (D1) and distance 2 (D2) as shown in **Figure 1**), 4) ‘*Time*’ (three levels – 0.5, 4 and 24 hours for BB and two levels – 0.5 and 24 hours for MRT); and 5) ‘*p53 status*’ (two levels, WT and KO). This classification produced 40 experimental groups. JCountPro software was used for preliminary analysis including classification of images and calculation of weighted average ± SE values for foci count per cell for each group. The weight for each image was assumed to be proportional to the number of counted cells per image. Standard error values reflected the experimental errors associated with inter image variability. The total number of counted cells per group varied from 470 to approximately 3000. JCountPro was also used to generate a set of data records for each individual image that included, apart from information on variables, the results of the image and counting analysis, such as the mean foci number per cell (fpc), number of cells in the image, cell area, cell and focus average intensity, etc. This data set incorporated entries for 364 analysed images.

We used this dataset to identify variables (factors) that significantly influenced the mean number of fpc. We applied the linear model generated using R language and tested a few models with various combinations of variables. We used Akaike’s Information Criterion (AIC) to evaluate models and Likelihood Ration Test to compare the models.

## Results

### Dosimetry

We estimated the doses of scattered radiation based on the measurements and approach reported previously (11, 12) for a similar irradiation setup. We assumed that the scattered dose is proportional to the geometrical area and the dose in the irradiated zone and the following range of distances from the nearest edge of the irradiated field: 2 - 12 mm and 10 – 18 mm for D1 and D2 groups respectively (**Figure 1**). We obtained the following dose values (mean ± SD): for BB D1, 200 ± 85 mGy (range 95 – 395 mGy); for BB D2 72 ± 20 mGy (range 50 – 120 mGy); for MRT D1, 24 ± 10 mGy (range 12 – 45 mGy); for MRT D2, 10 ± 3 mGy (range 6 – 15 mGy).

### *γ*-H2AX Response Generated by Scattered Radiation and Bystander Signalling

**Figure 2:**
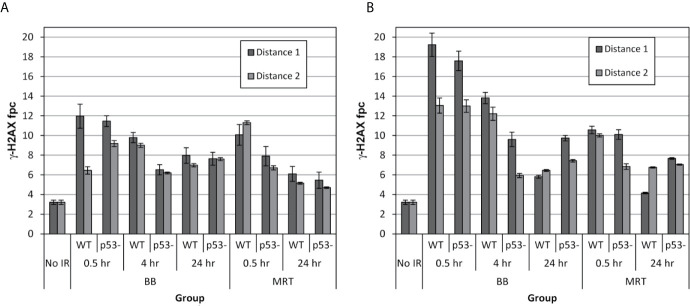
Mean numbers of *γ*-H2AX fpc induced in well 1 **(A)** and well 2 **(B)** following irradiation of the targeted field with 40-Gy synchrotron BB and MRT pulse in p53-WT and p53-null HCT-116 cells, at two distances from the irradiated field. An 8x8 mm zone of well 2 was irradiated, therefore the non-targeted cells in well 2 were exposed to factors emitted by the irradiated cells. Well 1 was physically separated from the irradiated field in well 2, therefore the cells in well 1were not exposed to the factors emitted by irradiated cells. After irradiation, the cells were fixed at noted times and processed for immunostaining. The values are the mean number of foci per cell ± standard error calculated from inter image variability.

The mean counts of *γ*-H2AX fpc in each experimental group, in p53-WT and p53-null HCT116 cells, are presented in **Figure 2** for well 1 (scatter) and well 2 (scatter and RIBE), and for two distances from the irradiated site, D1 (adjacent to the irradiated site), and D2 (adjacent to D1, as shown in **Figure 1**).

Scattered radiation (**Figure 2A**) generated by both BB and MRT induced significant DNA damage in both p53-WT and p53-null cell lines at both distances. The maximum extent of DNA damage was the highest at 0.5 hours post-irradiation which is consistent with well-described *γ*-H2AX response in directly irradiated cells (23, 32). Interestingly however, although diminished at later time-points, under all variable conditions at 24 hours post-irradiation the residual numbers of foci were substantially higher than the values prior to irradiation. From our earlier study (23), the *γ*-H2AX response of HCT-116 cells to 2.5-Gy conventional X-ray radiation was common for targeted cells, where most of DNA damage was efficiently repaired by 24 hours post-irradiation. It has been shown that prolonged maintaining of unrepaired DNA damage is a signature of RIBE (20). Therefore, DDR in well 1, generated by scattered radiation can be described as a mixed response of irradiated and bystander cells.

*γ*-H2AX foci counts in well 2 are presented in **Figure 2B**. In this well, irradiated cells in the top left corner (area 8x8 mm) shared medium with the cells that were not directly irradiated, but received the same range doses of scattered irradiation as cells in well 1. In addition, the cells in well 2 were exposed to the signalling from the irradiated cells. We hypothesised that by subtracting the scatter-generated foci values scored in well 1 from the both scatter and RIBE-generated values scored in well 2, we would be able to calculate the true RIBE-generated DNA damage in non-targeted cells, and therefore will be able to quantitate the input of scattered radiation in generation of DNA damage in non-targeted cells. We expected that due to contribution of an additional factor, communication between irradiated cells and non-irradiated neighbours, the DNA damage in non-targeted cells would be higher in well 2 than in well 1. Indeed, the early induction of *γ*-H2AX foci in well 2 at similar distances from the irradiated area was substantially higher (almost 2-fold) in BB-irradiated cells compared to well 1. However, it was not the case for MRT-irradiated cells. Quantitative analysis of the results is presented in the next section.

### Analysis of the *γ*-H2AX Response to Synchrotron Radiation

**Figure 3:**
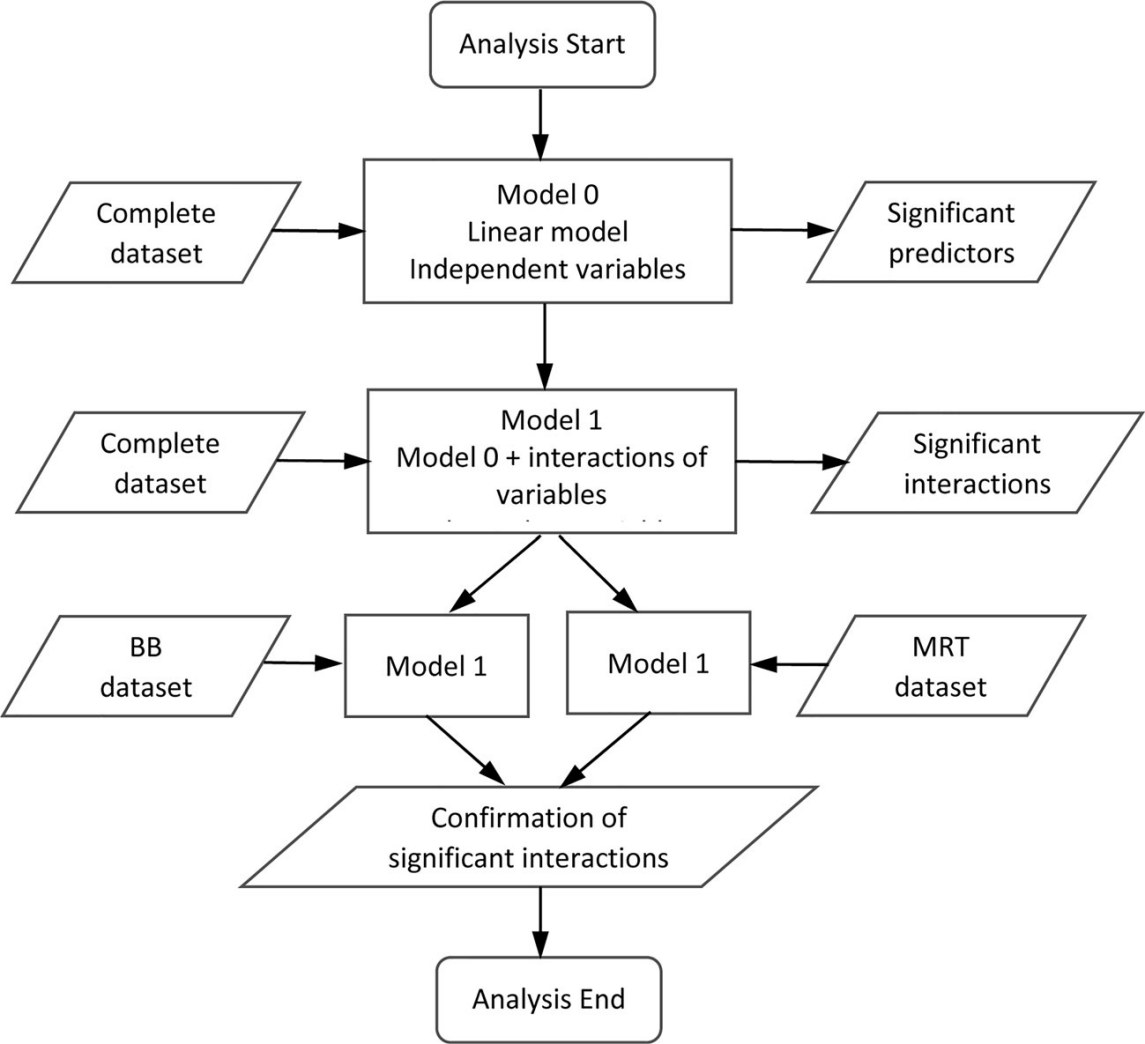
Flow chart illustrating the sequence and logic of the analysis of the *γ*-H2AX response. The flow chart maps out the steps taken to the modelling approach to test various assumptions and comparisons of the foci datasets analyzed in **Tables 1**, **2** and outlined in the text.

To evaluate the impact of various factors on the measured fpc numbers, we used the linear statistical model that was applied for the data set of 367 images described in *Materials and Methods*, considering the mean fpc number as the cellular response end-point. The sequence and logic of our analysis is illustrated by a flow chart in **Figure 3**. We initially generated a model that included the following factors: ‘*Beam’, ‘Well’, ‘Distance’, ‘Time’, ‘p53 status’*, and considered these factors as independent categorical variables. There are three levels for ‘*Beam’* (no beam, BB, MRT) and ‘*Time’* (0.5 *h*, 4 h, 24 h), and two levels for ‘*Well’* (Well 1, Well 2), ‘*Distance’* (D1, D2) and ‘*p53 status’* (WT, null). The first listed level for each variable was considered as a zero (baseline) level relative to which the effect of variables was estimated. The results of this analysis shown in **Table 1** (Model 0) indicated that all these factors, except ‘*p53 status’*, have significant impact on the cellular response. As the next step, we included in Model 0 interactions between different two variables and found that interactions ‘*Well*/*Time’*, ‘*p53 status*/*Time’* and ‘*Beam*/*Well’* had a significant impact on the cellular response (Model 1 in **Table 1**). Inclusion of these interactions significantly improved the model, as indicated by the decreased value of AIC and p<0.001 for likelihood ratio test, and did not affect the significance of individual factors.

**Table 1:** Comparison of two linear models of response (mean foci per cell count) and statistical significance of response predictors.

Variables (Levels)	Model 0	Model 1
*Beam* (BB/MRT)	***/***	***/***
*Well* (Well 2)	***	***
*Distance* (D2)	***	***
*p53 status* (null)	ns	ns
*Time* (4 h/24 h)	***/***	ns/***
*Well* (Well 2)/*Time (*4 h/24 h)	na	**/***
*p53 status* (null)/*Time (*4 h/24 h)	na	ns/**
*Beam* (BB)*/Well* (Well 2)	na	*
AIC – Akaike’s Information Criterion	1993.7	1941.0
χ2 – chi squared statistics/difference in degrees of freedom for Likelihood Ratio Test (LRT)		62.7/5
p – value (LRT)		<0.001

In parenthesis, levels of variables are indicated for which statistical significance of effect was calculated relative to the baseline, as defined in the text. Statistical significance codes: ***p < 0.001, **p < 0.01, *p < 0.05, ns - p > 0.05, na – not applicable.

**Table 2** summarises parameter estimates for factors used to predict the mean fpc number that provides an indication of the impact of individual factors and their interaction. A positive value of ‘*Intercept’* (3.21 ± 0.86, p = 0.00021) shows the background fpc number. Substantial positive values for ‘*Beam’* (7.13 ± 1.02, p < 0.0001 for BB and 5.57 ± 1.09, p < 0.0001 for MRT) indicate significant induction of DNA damage by scattered radiation at 0.5 hours in WT cells, that is more efficient for BB than for MRT. A negative parameter value for ‘*Distance’* (-1.23 ± 0.32, p = 0.00013) reflects the expected effect of lower scatter doses at D2 compared to D1. The role of RIBE in the induction of DNA damage is illustrated by a substantial positive value of the parameter estimate for ‘*Well’* (3.39 ± 0.71, p < 0.0001). A negative value for ‘*p53 status’* (-0.97 ± 0.55) shows a trend for the reduced response in p53-null cells, however this result is not statistically significant (p = 0.075). The overall impact of ‘*Time’* is not statistically significant at 4 hours (p = 0.661) and results in the reduced response at 24 hours (-2.97 ± 0.66, p < 0.0001), reflecting DNA DSB repair.

**Table 2:** Parameter estimates for factors used to predict the response.

Variables (Levels)	Estimate (*β*)	Std Error *β*	p-value
Intercept	3.21	0.86	0.00021
Beam (BB)	7.13	1.02	1.5e-11
Beam (MRT)	5.57	1.09	5.35e-7
Well (Well 2)	3.39	0.71	2.85e-6
Distance (D2)	-1.23	0.32	0.000126
p53 status (null)	-0.97	0.55	0.075
Time (4 h)	0.369	0.84	0.661
Time (24 h)	-2.97	0.66	7.99e-6
Well (Well 2)/Time (4 h)	-2.66	1.00	0.0080
Well (Well 2)/Time (24 h)	-4.04	0.72	3.14e-8
p53 status (null)/Time (4h)	-1.86	0.97	0.057
p53 status (null)/Time (24 h)	2.25	0.71	0.0016
Beam (BB)/Well (Well 2)	1.50	0.58	0.038

In parenthesis, levels of variables are indicated for which parameter estimates were calculated relative to the baseline, as defined in the text.

Analysis of the interaction of factors shows a significant impact of ‘*Time’* for well 2, resulting in a reduced response for both 4 and 24 hours, and an increased response at 24 hours for p53-null group (2.25 ± 0.71, p = 0.0016), ie an effect of p53-null status on DNA repair.

An interesting observation is a parameter estimate for ‘*Beam/Well’* interaction factor (1.50 ± 0.78, p = 0.038) indicating a higher response in well 2 for BB exposure prompting an interpretation of reduced RIBE following MRT irradiation. To further clarify this question, we applied Model 1 (without ‘*Beam/Well’* interaction) separately to BB and MRT data subsets. The results indicated a significant positive impact of ‘*Well’* for the BB data subset (6.53 ± 0.67, p < 0.0001) and non-significant trend of ‘*Well’* impact for the MRT subset (0.84 ± 0.79, p = 0.29), thus supporting the higher RIBE following BB irradiation. The separate analysis of the ‘*Beam’* subsets revealed two more interesting observations. We did not find a statistically significant interaction between ‘*Beam’* and ‘*p53 status’* factors in Model 1 for complete data set, and the impact of ‘*p53 status’* was not significant for the BB subset (0.30 ± 0.74, p = 0.68). However, it was negative and statistically significant for the MRT subset (-2.24 ± 0.73, p = 0.00269). We suggest an interpretation that assumes the reduction by p53-null status of the response to scattered radiation, which is a major contributor in MRT case, but not to bystander signalling, which significantly contributes in BB case. We also found that the impact of ‘*Distance’* was not significant for the MRT subset (-0.17 ± 0.44, p = 0.70), while it remained negative and statistically significant for the BB subset (-1.97 ± 0.41, p < 0.0001),

### The *γ*-H2AX Response to Low Doses of Conventional X-Ray Irradiation

**Figure 4:**
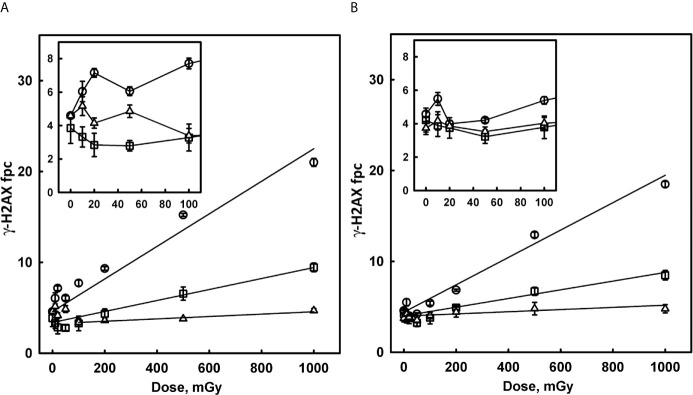
Induction of *γ*-H2AX foci in p53 WT **(A)** and p53-null HCT-116 cells **(B)** by low doses of X-rays. Cells were irradiated with various doses of 160 kVp X-rays and fixed 0.5 hours (circles), 4 hours (squares) and 24 hours (triangles) post-irradiation. Inserts in the top left corner of each panel show detailed pattern of the dose response in the region of small doses from 10 to 100 mGy. Solid lines in the main panels represent results of linear regression (ignoring 10 – 50 mGy data). Values of foci per cell yield per Gy (the slope of linear component) are as follows: 18.8 ± 1.4, 6.05 ± 0.74 and 1.31 ± 0.19 for p53 WT at 0.5, 4 and 24 hours respectively; 15.1 ± 1.3, 4.78 ± 0.65 and 1.20 ± 0.44 for p53-null at 0.5, 4 and 24 hours, respectively.

To better understand the radiation dose response of *γ*-H2AX foci induction by scattered radiation, we studied the response of HCT-116 cells to graded low doses of conventional X-rays (in the range from 10 to 1000 mGy). The results of this study are presented in **Figure 4** as a dose response of *γ*-H2AX fpc detected at 0.5, 4, and 24 hours post-irradiation for both p53 WT and p53-null cells. These results demonstrate some important features. The dose response is not linear, with a linear component that is only evident at doses above 100 mGy, and a non-linear component with a complex pattern at lower doses. The classical linear component is associated with induction of DNA DSB from clusters of ionisation and hydroxyl radical formed by irradiation in the vicinity of DNA. We calculated the yield of foci per Gy for each time point. The results of this calculation (shown in **Figure 4** legend) indicate a trend for a reduced response in p53-null cells compared to WT cells (15.1 versus 18.8) however the difference is not statistically significant (p = 0.093, df =7, two-sided test). DNA damage repair was substantial at 4 hours and 24 hours post-irradiation for both WT and p53-null cells.

The non-linear component, shown in the insets in **Figure 4**, which presumably reflects DNA damage from endogenous cellular factors induced by IR, follows a complex pattern. There is an increase in fpc at 0.5 hours for doses 10-20 mGy, which is more pronounced for WT cells, followed by a decrease in above 20 mGy region. At larger doses (>100 mGy), the contribution of the linear component becomes noticeable and dominant. Interestingly, the decrease is well pronounced at 4 hours, with fpc values below background values, presumably due to repair of the majority of induced DNA DSB, as well as the background DNA DSB. In summary, such complex dose response may be related to the activation of cellular competing, damaging and protecting mechanisms at various doses.

Our extended analysis (data not shown) also revealed over-dispersion of foci frequency distributions, as compared to the random (Poisson) statistics, since non-linear component effects are associated only with a subpopulation of cells with abnormally high foci numbers, while Poisson distribution is a feature of the linear component.

## Discussion

In this study we employed the *γ*-H2AX assay, a recognized biomarker of DNA damage and a well-established end-point for RIBE studies. In our earlier studies at the AS, detection of H2AX phosphorylation was utilized to trace the induction and repair of non-targeted DNA damage induced by synchrotron radiation *in vitro* (11) and *in vivo* (12–14). Post-irradiation kinetics of *γ*-H2AX foci formation and decline for targeted normal tissues are well-studied and dose-dependent. The maximum foci formation is detected within 15 min to 1 hour post-radiation exposure, peaking at about 1 - 2 hours followed by a progressive decline and returning to near-baseline levels by 24 hours post-exposure (18, 31, 32). *γ*-H2AX foci form to various extents in tumour cell lines, and generally follow the typical post-irradiation kinetics (38). Relevant to this study, earlier we have reported an efficient DNA damage repair in HCT-116 WT cell line (23).

The DNA damage response of bystander cells has several signature characteristics. The kinetics is distinctly different – commonly, *γ*-H2AX foci slowly accumulate and slowly disappear (20, 24). Under certain conditions, eg co-culturing of shielded (bystander) and unshielded (exposed) portions of sensitized HCT-116 cultures after exposure to UVA light to generate DSBs, a quick (30 min) but long-lasting bystander response was generated (23). In addition, highly proliferating and transcribing cells have been identified as the most vulnerable to bystander signalling (21). RIBE seems to be genetically controlled; eg DNA repair-deficient cells have been reported to produce bystander responses to a larger extent (39). Biological consequences of RIBE are cell type- and tissue-specific (40), and tumour cells are particularly susceptible (32).

Here we quantified *γ*-H2AX foci induced in non-irradiated cells at different distances from the 8x8-mm zone irradiated with BB-or MRT to a peak-dose of 40 Gy, and compared the values generated by scattered irradiation and by cell-cell communication in the irradiated and unirradiated wells. We detected that substantial numbers of *γ*-H2AX foci were induced by scattered radiation in well 1, that stayed elevated even at 24 hours post-irradiation. The long maintenance of unrepaired DNA damage after low-dose IR has been reported (41). We suggest an ongoing exchange of bystander signalling between cells exposed to low-dose scattered irradiation as an explanation of this observation in well 1. We detected even higher numbers of *γ*-H2AX foci in bystander cells in well 2, thus addressing the importance of the contribution of scatter radiation in studies of RIBE induced by partial irradiation of cell populations. We found a more pronounced bystander response following irradiation with BB compared to MRT. *γ*-H2AX foci numbers were generally dependent on the distance from the irradiated site at the earlier, but not at the later time-points, thus reflecting the scattered dose gradient in well 1 and the time-dependent transfer of bystander signals from cells that were exposed to targeted irradiation or scatter.

Considering the doses of scattered radiation in our bystander experiments, and based on the results of conventional X-ray irradiation, we can conclude that both linear and non-linear DSB induction components contribute to the measured foci number for broad beam scattered radiation (95–395 mGy for D1 and 50–120 mGy for D2), in contrast to the mainly non-linear component involved for MRT beam (12–45 mGy for D1 and 6–15 mGy for D2). The different roles of the two components to BB and MRT response are supported by the observation that the distance from the irradiated site is a significant factor for BB and not significant for MRT beam. The extent of response to scattered radiation is broadly consistent with conventional X-ray irradiation data, however large variability in scattered radiation doses obtained by individual cells in each group makes an accurate comparison quite difficult. Assuming that the shape of the non-linear dose response for X-rays is related to the activation of two competing mechanisms, the role and contribution of these mechanisms for scattered radiation is not clear, due to the scattered dose variability within an irradiated cell population.

We also addressed a question whether loss of p53 would modify DDR in non-irradiated cell cultures. More than 50% of human cancers carry mutations in this major tumour suppressor gene (42). The mutations are very diverse, with the vast majority resulting in loss of p53’s ability to bind DNA in a sequence-specific manner and activate transcription of canonical p53-target genes (43). Changes in its function along with other tumour suppressors/oncogenes lead to metabolic alterations necessary for tumour progression such as high rates of glycolysis, lactate production, biosynthesis of lipids and nucleotides, and the altered immune response (44, 45). p53 has a central role in DNA damage responses; it affects the cell’s ability to induce cell cycle arrest, senescence, apoptosis and DNA repair (46). It modulates homologous recombination (HR) by regulation of repair factors such as Rad51 and ATM/ATR, and has genetic interactions with components of non-homologous end joining (NHEJ) repair pathway (47). The HCT116 53-null cells from Vogelstein’s group used in this study were generated by sequential disruption of the two p53 alleles by two promoterless targeting vectors, each containing a geneticin- or hygromycin-resistance gene in place of genomic p53 sequences of the parental cell line (28). HCT116 p53-null cells, although they had a selective growth advantage, could not normally enter mitosis and replicate after being exposed to DNA-damaging agents, such as ionizing radiation (28). Investigations of a role of p53 in RIBE *in vitro* have been more or less consistent. In a study that employed the medium transfer protocol, both HCT-116 WT and p53-null cells have been found to induce RIBE, however there were variations in its extent for different end-points (viability, micronuclei, apoptosis and senescence) (48). Another medium-transfer study, by clonogenic survival end-point, has reported that both HCT-116 WT and p53-null cells produce bystander signals, but only p53 WT cells respond to the signals (49). The p53 status of bystander human lymphoblastoid cell lines sharing medium with irradiated cells was considered in the study of Zhang et al. (50), and no differences in bystander signal production/response measured by radiation mutagenesis were found. In *in vivo* model, when athymic female nude mice implanted with HCT-116 p53 WT and p53-null cells into both flanks, and only one flank was irradiated, the growth of non-irradiated WT tumours was inhibited to a larger extent (ie anti-tumour abscopal effect) due to the apoptotic pathway activation, compared to non-irradiated p53-null tumours (51).

In this study, a common trend for overall reduced response was observed to both scattered and conventional low-dose X-ray irradiation, but not to bystander signalling. The deviations from linearity for the low-dose response (**Figure 4**) may indicate the low-dose hyper-radiosensitivity (HRS) phenomenon. By monitoring single cell proliferation, cell cycle markers and apoptosis in tumour cell lines, including p53 WT and p53-null HCT116 cells, Enns et al. reported p53-dependent HRS (52). Studies focused on complex relationship between HRS/increased radioresistance at low doses and RIBE have been conducted in Mothersill’s group. Earlier work by Mothersill et al. on 13 tumour cell lines with or without p53 abnormalities, revealed that cell lines with large RIBE do not show HRS, without clear dependence on the p53 status (6). RIBE and HRS/radioresistance seemed to be mutually exclusive after irradiation with doses >1 Gy. A study by Fernandez-Palomo et al., by employing two tumour cell lines, one with a strong transition from HRS to induced radioresistance, and another that lacked HRS, suggested that cell killing in the HRS region can be associated with RIBE (53). The authors have shown that in the part of the survival curve showing HRS there was RIBE, but when the dose increased and the radioresistance portion of the survival curve was reached, RIBE was lost. In the clinical scenario, slower DNA damage repair in out-of-field tissues may cause tumour and normal tissue HRS to low-dose scattered radiation in individuals carrying p53 deficiencies.

Thus, the *γ*-H2AX end-point is a useful tool to follow spatio-temporal changes of scatter radiation- and RIBE-induced DNA damage in two colon carcinoma cell lines, WT and p53-deficient. For the first time, we showed that in RIBE studies that exploit a microbeam irradiation of a subcomponent of cultured cells, there is a substantial contribution of the scattered radiation to bystander DNA damage, that needs to be considered for the correct evaluation of RIBE. On the other hand, cells exposed to the low-dose scatter generate unrepairable DNA damage, possibly due to an ongoing exchange of bystander signalling. These results need to be taken into account for risk estimate of side effects when conducting synchrotron radiation experimentation on living biological targets and for cancer treatment in patients.

In conclusion, biological effects of FLASH-RT and MRT are a “hot topic” in current translational radiation research, as many studies have shown in animal models that both FLASH and MRT provide equivalent or better tumour control than conventional fractionated RT, with a major benefit being the significantly reduced damage to normal tissues within the field (29, 54, 55). Accordingly, here we report a more pronounced bystander response following irradiation with broad beam compared to MRT. Better understanding of non-targeted effects of novel radiation modalities will allow increasing the therapeutic ratio by minimising DNA damage in non-targeted normal tissues, as well as by contributing to development of strategies for enhancement of anti-tumour abscopal effects.

## Data Availability Statement

The raw data supporting the conclusions of this article will be made available by the authors, without undue reservation.

## Author Contributions

PL, HF, CS and OM contributed to conception and design of the study. OM and VD organized the funding. PL, HF, AI, JM, AS, CH, CS and OM conducted experiments. PL performed the statistical analysis. PL and OM wrote the first draft of the manuscript. All authors contributed to the article and approved the submitted version.

## Funding

This study was supported by the Australian National Health and Medical Research Council (NHMRC) grant 10275598, by the 2010 round of the priority-driven Collaborative Cancer Research Scheme (grant 1002743) and the Australian Government Department of Health and Ageing with the assistance of Cancer Australia. Support was also provided by the Victorian Government’s Operational Infrastructure Support Program and by Swiss Cancer Research foundation grant KFS-4281-08-2017.

## Conflict of Interest

The authors declare that the research was conducted in the absence of any commercial or financial relationships that could be construed as a potential conflict of interest.
